# Examining The Effects of Silver Diamine Fluoride Treatment on the Surface Morphological Properties and Binding Strength of Demineralized Dentin to Glass Ionomer with and without Potassium Iodide and Glutathione: Examining The Effects of Silver Diamine Fluoride Treatment on the Surface Morphological Properties

**DOI:** 10.31661/gmj.v13iSP1.3555

**Published:** 2024-12-31

**Authors:** Mahsa Samani, Farimah Hajebi, Faramarz Zakavi, Mehran Mapar

**Affiliations:** ^1^ Department of Restorative Dentistry, School of Dentistry, Ahvaz Jundishapur University of Medical Sciences, Ahvaz, Iran; ^2^ Department of Aesthetic and Restorative Dentistry, School of Dentistry, Iran University of Medical Sciences, Tehran, Iran

**Keywords:** Silver Diamine Fluoride, Potassium Iodide, Glutathione, Microtensile Bond Strength

## Abstract

One new and promising chemical for non-invasive and minimally invasive dental caries treatment is silver diamine fluoride (SDF). However, demineralized dentin discolors easily, so it’s not a popular choice for permanent teeth or the aesthetic zone. Although this discolouration can be reduced by applying glutathione and potassium iodide (KI) antioxidants, it is unclear how these substances affect the bond strength of glass ionomer (GI) to pre-treated dentin. Consequently, the objective of this research was to evaluate the degree to which dentin treated with SDF plus KI (SDF-KI), SDF plus glutathione (SDF-GLU), or a combination of the two (SDF+GLU) compared to GI in terms of microtensile bond strength (mTBS). We artificially demineralized 75 dentin specimens taken from healthy human permanent teeth to mimic caries. After that, they were connected to self-cure GC-Fuji IX GI and treated in one of five groups: control, SDF, SDF-KI, SDF-GLU, or SDF+GLU (n=15). Their mode of failure was identified under a stereomicroscope after they went through a mTBS test. Scanning electron microscopy (SEM) was also applied to a subset of specimens from both groups. The control and SDF-KI groups had significantly different mTBS values (P=0.019 and P=0.005, respectively), as did the control and SDF-GLU groups. Compared to the SDF group, the SDF-KI group had a significantly lower mTBS (P=0.024). There was a statistically significant difference between the SDF and SDF-GLU groups in terms of mTBS (P=0.006). When comparing the control group to the SDF and SDF+GLU groups, there was no significant difference in mTBS (P0.05). For optimal dentin surface preparation prior to GI restoration, SDF+GLU is the way to go. The decrease in GI to dentin mTBS is the reason why SDF-KI and SDF-GLU are not suggested.

## Introduction

The current literature holds that dental caries is more of a behavioral illness involving bacteria than an infectious one. Cavitated dental caries treatment no longer involves cavity preparation by removing all infected and damaged dentin and expanding the cavity to accommodate the carious lesion’s size [1]. Atraumatic restorative techniques (ARTs) have been used in distant communities as a conservative caries control approach for years [2]. However, non-invasive dentistry has received more attention in recent years, compared to before [3][4]. Thus, the indications of ARTs have increased, and ARTs are no longer limited to deprived and distant areas; they are currently practiced in many dental clinics in modern countries as a standard treatment approach, particularly for the elderly and non-cooperative patients, and those with special needs [5][6].

Glass ionomer (GI) cement is a restorative material with fluoride release potential, which is commonly used in ARTs[7][8]. It should be recharged by a source of fluoride for optimal efficacy[9]. Silver diamine fluoride (SDF) is a bactericidal, colorless, and low-cost material, which forms CaF2-like compounds in reaction with dentin hydroxyapatite that serves as a slow-release fluoride reservoir. The presence of fluoride and silver together in an alkaline environment has a synergistic effect on the cessation and control of dentin caries[10][11]. Thus, SDF is superior to other fluoride-containing compounds. There is no need to remove the soft carious tissue before the application of SDF[12]. Also, the application of SDF is more effective than the temporary restoration of the cavity with GI to stop the progression of cavitated caries[13]. Based on this, SDF is compatible with ARTs. The silver-modified atraumatic restorative technique (SMART) is the name given to the combination of SDF and an ART [14]. By using SMART, pulp vitality may be maintained, carious lesions can be remineralized, and secondary caries surrounding GI restorations can be prevented [15][16]. SMART significantly increases the concentration of released fluoride and results in more successful caries management [17].

SDF can also be used as a liner beneath restorations[16], in the treatment of root caries in the elderly, medically compromised patients, and those with behavioral problems, and in the restoration of caries in primary teeth of children who are candidates for dental treatment under general anesthesia but cannot or are not willing to do it[18][19][20]. In all such cases, the tooth treated with SDF should be restored to resume its function or for aesthetic reasons. GI is the first restorative material selected for this purpose. Thus, it is important to ensure no adverse effect of treatment with SDF on the quality of the bond of GI to dentin, to guarantee the success of dental restorative procedures with SDF[21].

SDF forms Ag3PO4 following a reaction with hydroxyapatite, which causes tooth discoloration[22]. Despite the extensive advantages of SDF, this discoloration limits its application in the esthetic zone and also in adults, such that except for root caries in the elderly, SDF has no other indications in adults[23][24]. A few methods have been suggested to lessen this coloration and make the treatment more patient-friendly [25], such as adding a super-saturated potassium iodide (KI) solution right after SDF is applied [which forms silver iodide (AgI) as a creamy white substance [26], using ammonium hexafluorosilicate or silicon fluoride instead of silver ions, or using silver nanoparticles [27][28].

Glutathione is a cross-linker with anti-oxidant properties, which is extensively produced in the human liver[29]. Cross-linkers interact with the extracellular components of the dentin matrix and form inter- and intra-molecular cross-links leading to dentin biomodification. Resultantly, some areas are formed in the cross-linked matrix where susceptible collagen fibers are protected against the effects of collagenases, and the mineral phase present in gaps between the collagen fibers is protected against further dissolution. Resultantly, the biomechanical and biochemical properties of dentin improve [30][31].

A decrease in glutathione levels and an increase in oxidative stress, or lipid peroxidation, can lead to a decrease in antioxidant properties, cell death, and inflammation when there are high levels of reactive silver ions in SDF. These cytotoxic effects on the dentin-pulp complex can also cause discoloration [32]. Therefore, there are some concerns about using SDF in deep cavities since it is cytotoxic to dental pulp cells [27]. One crucial component in preventing silver cytotoxicity is thiol-containing compounds (NACs) like glutathione, which can neutralize the action of silver ions [33]. Glutathione can also form a coating around silver particles and minimize discoloration in teeth treated with SDF by reducing of accumulation of silver particles and controlling the speed of dispersion of silver ions[34]. Some concerns exist regarding the reduction in the number of free silver ions and the subsequent reduction in the beneficial effects of SDF in the use of KI[35].Other possible side effects of KI application include the possibility of adverse effects on the fetus in pregnant women, interference with thyroid function, eliciting allergic reactions, edema in salivary glands, and soft tissue desquamation[36].

Chelation of hydroxyapatite, which occurs when polyacrylate ions react with calcium in its structure, is responsible for GI cement’s adherence to dentin. Cement adherence is influenced by the calcium content of the tooth structure [37]. In contrast, SDF polishes teeth by raising their surface calcium and phosphorus levels. Also, amorphous calcium phosphate, which is derived from hydroxyapatite, is generated, and its calcium to phosphorous ratio can be adjusted [38].In addition, silver ions in SDF can partially substitute for calcium ions in hydroxyapatite, resulting in the formation of silver hydroxyapatite [11].This suggests that SDF may have the ability to modify the GI bond strength to tooth structure [39]. The findings on this subject have sparked debate. While some research found that SDF was compatible with GI restorations, other studies found that it weakened the bond strength between GI and dentin when applied [40].When it comes to improving the bond strength of GI to dentin that has been prepared with SDF and KI, or to masking the discolouration, there are a number of research that you can refer to. Glutathione has been shown to be effective in reducing staining[24], but no one has yet tested how combining glutathione and SDF affects the bond strength of GI. Its wide-ranging dental use necessitates supplementary studies comparing SDF-GLU, SDF-KI, and SDF independently. Consequently, the purpose of this research was to evaluate and contrast the microtensile bond strength (mTBS) of demineralized dentin that had been treated with SDF, KI, and glutathione, as well as dentin that had not been treated (control group).

## Materials and Methods

From dental clinics, researchers gathered 75 healthy molars that were free of restorations, cracks, and cavities for this investigation. No teeth were pulled for this study, as the teeth that were extracted were due to periodontal disease (IR.AJUMS.REC.1400.692). The teeth were placed in a solution containing 0.1 weight percent thymol as soon as they were extracted. Within six months following extraction, the teeth were put to their full potential. The Ahvaz Jundishapur University of Medical Sciences ethics committee gave its stamp of approval to the study (The ethical code is IR.AJUMS.REC.1400.692). Acrylic resin was used to install the teeth, up to 1 mm below the cementoenamel junction. To reveal the intermediate dentin, a low-speed diamond saw was used to remove the occlusal enamel. The next step was a 10-second water polish using 600-grit silicon carbide abrasive paper. Dentin demineralization was mimicked by immersing the teeth in a demineralizing solution (500 mM acetate, 2.2 mM potassium dihydrogen phosphate, and 2.2 mM calcium chloride) at 37°C for 7 days. The demineralized dentin layer was removed and a typical smear layer was created by gently polishing the teeth with 600-grit silicon carbide abrasive paper. There was a single specimen prepared from each tooth. Here is how the specimens were divided into five groups, each with fifteen specimens:

Group 1 (control): Dentin surface was rinsed with saline alone.

Group 2 (SDF): Dentin was treated with a single drop of SDF (Cariestop 38%, Biodinamica, Brazil) using a microbrush. After one minute, the area was washed for thirty seconds.

Section 3 (SDF-KI): One minute following the SDF application, a microbrush was used to apply KI (Merck, Germany) to the dentin surface in order to get a creamy color deposit. A 30-second rinsing of the dentin followed.

Group 4 (SDF + GLU): The procedure was similar to group 2, with the difference that instead of SDF, a mixture of SDF and glutathione (Glutathione reduced; Merck, Germany) was applied.

Group 5 (SDF-GLU): The procedure was similar to group 3, with the difference that instead of KI, 20% glutathione was used after the application of SDF.

Tygon tubes with 1 mm length and 0.79 mm internal diameter were then placed on each dentin specimen and filled with self-cure restorative GI (Fuji IX, GC, Japan). After that, the Tygon tubes were separated from the attached GI cylinders using a surgical blade. The specimens were then placed in distilled water and incubated at 37°C for a whole day. A cyanoacrylate adhesive (Super adhesive; Razi, Iran) was used to attach the specimens to the testing jig after they were taken out of the incubator and allowed to air dry. Following the procedure of earlier research, the mTBS test was carried out. The cylinders were attached to a universal testing machine (AG-X plus, Shimadzu, Kyoto, Japan) using stainless steel wire loops (0.2 mm).

At the tooth-GI interface, the wire was looped and then spun around each GI cylinder. Pulling the wire parallel to the cylinder at a crosshead speed of 1 mm/minute applied a progressive stress until debonding occurred. After each of the three iterations, we took note of the average. The bond strength was determined in megapascals (MPa) by dividing the kilogramme debonding force by the cross-sectional area. A stereomicroscope (SZX16; Olympus, Tokyo, Japan) was used to examine the specimens after testing. The mode of failure was identified based on the proportion of GI that remained on the dentin surface. Based on the results, we can say that the failure mode was either cohesive within the GI or an adhesive at the GI-dentin interface. Following the bond strength test, the following was done to determine the failure mode in accordance with Khor et al:

• The failure rate of the adhesive between GIC and dentin ranged from 0% to 19%.

• A mixed failure occurred when 20-79% of the GIC failed.

• A cohesive failure occurred when 80-100% of the GIC failed.

After determining the mode of failure, the dentin part of broken specimens was scanned, and the most prominent region was recorded. The selected specimens were air-dried and mounted on brass pieces with carbon adhesive. They were gold sputter-coated with pure gold, placed on a special tray, transferred to a vacuum chamber, and inspected under a scanning electron microscope (SEM; VEG TESCAN-XMU, Czech Republic).

### Statistical Analysis

The Kolmogorov-Smirnov test was used to determine if the data followed a normal distribution. Data that followed a normal distribution were examined using two-way ANOVA and Tukey’s post hoc test. The Kruskal-Wallis and Mann-Whitney tests were used to assess data that did not follow a normal distribution. Statistics were performed using SPSS version 21 (SPSS Inc., IL, USA) with a significance level of 0.05 for all analyses.

## Results

**Table:** Table1. Comparing mean of bond strength(Mpa)in the five groups(control,SDF,KI,mix SDF and Glutathione and SDF+Glutathione) (n=15)

**Groups**	**Mean** **(MPa)**	**Sample size(N)**	**Std. Deviation**	**P-Value**
Control	19.5733	15	12.25870	
SDF	19.2327	15	10.04620	
SDF-KI	11.2473	15	4.58846	0.02
)SDF-GLU(	14.6220	15	9.66362	
SDF + GLU	9.5433	15	9.03194	
Total	14.8437	75	10.06847	

**Table:** Table2. Frequency of different modes of failure(cohesive,adhesive and mixed failure)in five groups of study

**GROUPS**		**FAILURE MODES (N)**		
	Cohesive	Adhesive	Mixed	**Total (n)**
CONTROL	3 20%	2 13.3%	10 66.7%	**15**
SDF	1 6.7%	2 13.3%	12 80%	**15**
SDF-KI	-	8 53.3%	7 46.7%	**15**
SDF+GLU	1 6.7%	6 40%	8 53.3%	**15**
SDF-GLU	-	9 60%	6 40%	**15**
	5	27	43	**75**

**KI:** Potassium iodide; **SDF:** Silver diamine fluoride; **GLU:** Glutathione

**Table:** Table3. Adhesive failure (GI-dentin interface) rates in five groups compared to one another: control, SDF, KI, mix SDF & Glutathione, and SDF+Glutathione.

	**Chi-Square Tests**		
	Value	Df	Asymptotic Significance (2-sided)
Likelihood Ratio	13.069	4	0.011
Pearson Chi-Square	12.970 ^a^	4	0.014
Linear-by-Linear Association	0.139	1	0.709
N of Valid Cases	75		

5 cells (50.0%) is expected count below five. The minimum expected count is 3.80.

### Microtensile Bond Strength Comparison

In this investigation, five sets of teeth were evaluated. The Kolmogorov-Smirnov test was used to determine if the data followed a normal distribution. Table-1 shows that there was a statistically significant difference in bond strength (Mpa) between all groups and the control group (P=0.02).

Following this, a post hoc test was used to compare all groups. The results showed that there were significant differences between the control and SDF-KI groups (P=0.019) and the control and SDF-GLU groups (P=0.005), as shown in Table-2.

Table-2 shows that compared to the SDF group, the SDF-KI group had a noticeably weaker mean bond strength to dentin (P=0.024). According to Table-2, the mean bond strength in the SDF-GLU group was also noticeably lower than in the SDF group (P=0.006). Concerning bond strength, though, SDF+GLU was ineffective. The SDF-KI group likewise had weaker bonds than the SDF+GLU group.

### Failure Mode Observation

Table-2 shows that only the control, SDF, and SDF+GLU groups showed signs of cohesive failure in GI. The frequency of adhesive failure varied significantly among the groups, according to statistical analysis (P<0.05). Mixed and cohesive failure rates were not significantly different across groups (P>0.05) as compared to the SDF+GLU group (Table-3).

### SEM Assessment

**Figure-1:**
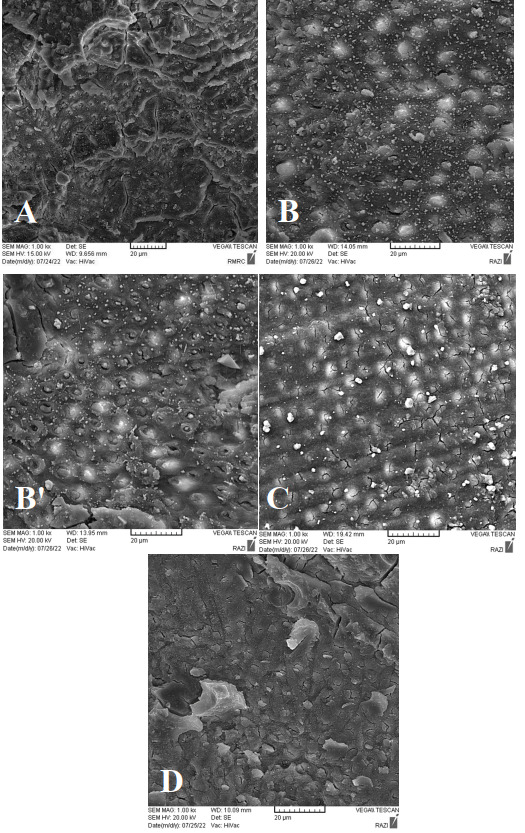
Figure 1. (A) failure mode of dentine-glass ionomer under scanning electron microscopy (silver diamine flouride group), (B) failure mode of dentine-glass ionomer under scanning electron microscopy (mixture of silver diamine flouride and glutathione group), (B’) failure mode of dentine-glass ionomer under scanning electron microscopy (mixture of silver diamine flouride and glutathione group)(C) failure mode of dentine-glass ionomer under scanning electron microscopy (silver diamine flouride then application of glutathione group), (D)failure mode of dentine-glass ionomer under scanning electron microscopy (silver diamone flouride then application of iodide potassium group)

Since high-viscosity GI cement contains water, their inspection under an SEM was difficult because the preparation of specimens through dehydration and metallization can cause artificial microcracks both within the material and at the bonding interface. Figures-1A to 1D show SEM micrographs of the study groups.

## Discussion

In this investigation, we aimed to find out whether adding SDF to demineralized dentin before bonding with GI weakens the bond strength, both with and without potassium iodide and glutathione. Dentin surfaces are coated with CaF2 globular particles and Ag3PO4 cubic crystals as a consequence of the reaction between SDF and tooth hydroxyapatite. CaF2 globular particles are eliminated by rinsing while Ag3PO4 cubic crystals are not dissolved by rinsing and undergo discoloration over time[41].Thus, SDF causes dark spots on the tooth structure, which create some concerns regarding its clinical use[42][43]. This discoloration limits the application of SDF in permanent dentition especially in the esthetic zone even if its definite cariostatic efficacy is confirmed[23].

Several studies have shown that SDF can be used with GI restorations without any problems. Nevertheless, there is a lack of research on the effectiveness of additives like SDF, KI, and glutathione in improving the bond strength between GI and demineralized dentin or in masking discolouration. The purpose of this research was to determine how well SDF, KI, and glutathione strengthened the link between GI and dentin. The affected dentin is partially demineralized and has adequate collagen fibers for remineralization. Therefore, according to the principles of minimally invasive dentistry, the affected dentin should be preserved clinically. The bond strength to damaged dentin is lower than to sound dentin, making bonding to this substrate hard. Artificial demineralization of dentin to mimic caries was thus performed in a controlled in vitro setting for this study [16][44]. Restoration of teeth treated with SDF to restore their function and improve esthetics is a challenge in restorative dentistry[45]. Fuji IX was selected as the restorative material for the present study because it is among the strongest restorative conventional GI cement available in the market, and has also been recommended by the WHO for ARTs[46].

When comparing the experimental groups to the control group, statistical analyses revealed that all of them had lower mean bond strengths to dentin. However, only the SDF-KI and SDF-GLU groups showed a statistically significant decrease in bond strength to dentin; applying SDF alone or SDF+GLU had no discernible effect on the bond strength of GI to demineralized dentin. The decrease in mean bond strength could be due to the silver in SDF reacting with protein phosphate groups, instead of reacting with the calcium in GI, which is present in the composition [46]. Consequently, one study found that SDF reduced the bond strength of GI to dentin[51], while another systematic review by Frohlich et al.[47] and other studies revealed that SDF had no effect on the link between GI and dentin[16][39][48][49][50]. Following a procedure similar to that of Knight et al., the current study applied SDF and then washed off the deposit [26].

A creamy white deposit of silver iodide is formed with application of KI saturated solution followed by SDF. This deposit limits the availability of silver ions, which are responsible for dark dentin discolouration, and hence avoids discoloration[30][52]. Having said that, the impact will fade with time[41]. Still, the present investigation found that the SDF-KI group had substantially weaker GI-dentin bond strength compared to the SDF group. The bond strength of GI to dentin was not significantly affected by applying KI solution to dentin after SDF treatment, according to Zhao et al. and Knight et al. The use of a macro-shear bond strength test, however, casts doubt on the reliability of the results. Even if they didn’t reveal how they used SDF in their research. Consequently, each of the aforementioned investigations have a substantial risk of bias[16][26][53]. François et al. and Knight et al. both found that GI bond strength to SDF-treated dentin was lowered when KI was applied, which is in line with the current findings[26][50][54].

In the case of KI, it is difficult to remove the silver deposits that are generated on the surface of the dentin. To remove the silver contamination on the surface and its negative impact on the bond strength between GI and SDF-KI-treated dentin, an extra layer of surface treatment with polyalkenoic acid is applied [50].

Before the application of high-viscosity GI cement, the use of polyacrylic acid and subsequent irrigation of dentin can improve the bonding quality. It partially demineralizes the dentin surface and enhances the possibility of chemical and micromechanical interactions between GI cement and hydroxyapatite[55]. Although the dentin conditioning protocol for FUJI IX GI includes the application of a 10% polyacrylic acid conditioner on the dentin surface before bonding, the use of phosphoric acid can improve the bond strength by eliminating of superficial biological impurities, the smear layer, and smear plugs[56][57].

Removal of the smear layer from the dentin surface by a conditioner such as polyacrylic acid can affect the bond strength[58]. However, in the present study, only the deposits caused by the reaction of SDF with KI were rinsed after the application of SDF-KI, and cleaning of the dentin surface with polyacrylic or phosphoric acid was not performed. This led to a dramatic weakening of the GI-dentin connection after treatment with SDF-KI. Before GI bonding, it seems that the dentin surface must be thoroughly cleaned of deposits that have been generated by the reaction of SDF-KI [59]. More long-term research comparing the properties and effects of SDF-KI for general usage throughout time are needed, according to the present study and earlier investigations [60].

A biomimetic coating of glutathione on silver particles has been employed in the past to improve their water solubility and their interactions with intricate biological systems. This compound controls the speed of release of silver ions and decelerates the process of discoloration of teeth treated with SDF over time[34].

Consistent with the findings of Priya et al., the present investigation found that SDF+GLU had no effect on binding strength. This discovery could be explained by the fact that glutathione, an antioxidant with properties that cross-link and stabilize collagen, can potentially enhance the binding strength in the SDF+GLU group [61]. Previous studies have demonstrated that even after rinsing off the SDF from the dentin surface, mineral deposits still form on the dentin surface and even within the dentinal tubules. A difference exists in this regard between the application of glutathione after SDF or mixing the glutathione with SDF and the application of the mixture on the dentin surface. Accordingly, when glutathione is used in combination with SDF, deposit formation on the dentin surface changes, and when glutathione is applied after the SDF, the formation of deposits significantly decreases. Also, due to no penetration of deposits, the opening of many dentinal tubules remains open; whereas, when glutathione is mixed with SDF, greater amounts of mineral deposits form on the dentin surface[62].

Using scanning electron micrographs (SEMs), the current investigation showed that dentin surface mineral deposits were plentiful when SDF mixed with glutathione (SDF+GLU) was applied (Figures-1B and 1B’). Due to the small size of deposits, they did not cause complete obstruction of dentinal tubule openings, enabling micromechanical, and to a lesser extent, chemical retention within the dentinal tubules. Moreover, due to the higher cross-sectional area of mineral deposits, compared with other groups, a stronger bond to GI was achieved in this group. However, in the application of glutathione after SDF (SDF-GLU), a cross-section almost free from mineral deposits (resembling an etched dentin surface) was observed (Figure-1C). Glutathione has an acidic pH, and materials with a low pH have a demineralizing effect on dentin and impair the stability of crystalline deposits on the dentin surface. This may be another reason explaining the elimination of mineral deposits from the dentin surface in SDF-GLU group after the application of 20% glutathione following the use of SDF on dentin[62][63].

In bonding to GI, the increased mineral content of dentin surface by the application of solutions that result in the deposition of calcium phosphate or calcium sulfate can increase the reactivity of the superficial layer with poly-acid[64][65]. The present study’s SEM micrographs, in conjunction with those of Kim et al.[62], suggest that the SDF+GLU group had the strongest bonds, while the SDF-GLU group had the weakest. This makes sense, given that the mineral content of the tooth structure determines the mechanism of GI bonding [55]. The reason may be that a previous study[62] showed that the abovementioned groups had the highest and lowest mineral deposition on the dentin surface, respectively. The present investigation found a similar pattern, with GI bond strength significantly reduced after dentin was pretreated with SDF-GLU. Despite the numerous advantages of SDF, it inhibits the activity of alkaline phosphatase, while this enzyme is imperative for mineralization and formation of tertiary dentin[66]. However, previous studies showed that the application of glutathione can inhibit the reduction in the level of this enzyme. On the other hand, SDF is toxic for dental pulp stem cells even in low concentrations (< 0.001%), and can cause their death[67]. On the other hand, glutathione is an antioxidant that has been proven to be effective in the reduction of cytotoxicity of SDF. Restorative procedures in permanent dentition, specifically in cavities near the pulp, and indirect pulp capping may benefit from the use of SDF+GLU because it can reduce discoloration caused by SDF and does not weaken the bond strength of GI to demineralized dentin, two of its main drawbacks [62]. To determine whether this molecule is harmful to pulp cells and whether glutathione modifies SDF’s ability to induce dentin remineralization, further research is needed.

## Conclusion

The application of SDF+GLU is a suitable method for dentin surface preparation before GI restoration. However, SDF-KI and SDF-GLU are not recommended due to the reduction in µTBS of GI to dentin.

## Conflict of Interest

The authors declare that they have no Conflicts of Interests.
